# Multiple Wnts Redundantly Control Polarity Orientation in *Caenorhabditis elegans* Epithelial Stem Cells

**DOI:** 10.1371/journal.pgen.1002308

**Published:** 2011-10-13

**Authors:** Yuko Yamamoto, Hisako Takeshita, Hitoshi Sawa

**Affiliations:** 1 Laboratory for Cell Fate Decision, RIKEN, Center for Developmental Biology, Kobe, Japan; 2 Department of Biology, Graduate School of Science, Kobe University, Kobe, Japan; 3 Multicellular Organization Laboratory, National Institute of Genetics, Mishima, Japan; University of California San Diego, United States of America

## Abstract

During development, cell polarization is often coordinated to harmonize tissue patterning and morphogenesis. However, how extrinsic signals synchronize cell polarization is not understood. In *Caenorhabditis elegans*, most mitotic cells are polarized along the anterior-posterior axis and divide asymmetrically. Although this process is regulated by a Wnt-signaling pathway, Wnts functioning in cell polarity have been demonstrated in only a few cells. We analyzed how Wnts control cell polarity, using compound Wnt mutants, including animals with mutations in all five Wnt genes. We found that somatic gonadal precursor cells (SGPs) are properly polarized and oriented in quintuple Wnt mutants, suggesting Wnts are dispensable for the SGPs' polarity, which instead requires signals from the germ cells. Thus, signals from the germ cells organize the *C. elegans* somatic gonad. In contrast, in compound but not single Wnt mutants, most of the six seam cells, V1–V6 (which are epithelial stem cells), retain their polarization, but their polar orientation becomes random, indicating that it is redundantly regulated by multiple Wnt genes. In contrast, in animals in which the functions of three Wnt receptors (LIN-17, MOM-5, and CAM-1) are disrupted—the stem cells are not polarized and divide symmetrically—suggesting that the Wnt receptors are essential for generating polarity and that they function even in the absence of Wnts. All the seam cells except V5 were polarized properly by a single Wnt gene expressed at the cell's anterior or posterior. The ectopic expression of posteriorly expressed Wnts in an anterior region and vice versa rescued polarity defects in compound Wnt mutants, raising two possibilities: one, Wnts permissively control the orientation of polarity; or two, Wnt functions are instructive, but which orientation they specify is determined by the cells that express them. Our results provide a paradigm for understanding how cell polarity is coordinated by extrinsic signals.

## Author Summary

Proper functions and development of organs often require the synchronized polarization of entire cell groups. How cells coordinate their polarity is poorly understood. One plausible model is that individual cells recognize extrinsic signal gradients that orient their polarity, although this has not been shown in any organism. In particular, although Wnt signaling is important for cell polarization, and Wnt signal gradients are important for the coordinated specification of cell fates, the Wnts' involvement in orienting cell polarity is unclear. In the nematode *Caenorhabditis elegans*, most asymmetrically dividing mitotic cells are polarized in the same anterior-posterior orientation. Here we show that multiple Wnt proteins redundantly control the proper orientation of cell polarity, but not for polarization per se, in a group of epithelial stem cells. In contrast, Wnt receptors are indispensable for cells to adopt a polarized phenotype. Most stem cells are properly oriented by Wnt genes that are expressed either at their anterior or posterior side. Surprisingly, Wnt signals can properly orient stem cell polarity, even when their source is changed from anterior to posterior or vice versa. Our results suggest the presence of novel mechanisms by which Wnt genes orient cell polarity.

## Introduction

For tissues and organs to be properly organized, it is often essential that cell polarity be coordinated among cell groups. In the *Drosophila* wing, for example, cells are polarized in the same proximal-to-distal orientation to produce hairs pointing distally [1]. Similarly, in the mammalian cochlea, stereociliary bundles form at the outer edge of all hair-producing cells [2]. Such coordinated polarizations are often controlled by the Wnt/PCP (planar cell polarity) pathway, which involves the polarized localization of signaling molecules such as Frizzled, Dvl/Dishevelled, and Van Gogh proteins [3]–[5]. One plausible model for cell polarity coordination is that individual cells recognize extrinsic cues that orient their polarity. Although Wnt proteins have been considered candidates for orienting molecules, their functions in regulating cell polarity are not well understood.

In *Drosophila*, where PCP phenotypes are lacking in some Wnt mutants, including *wingless*, Wnt proteins are not believed to be required for regulating PCP. Instead, PCP is coordinated by communication between neighboring cells, although the presence of extrinsic cues is still anticipated. In the mammalian cochlea, Wnt7a has been suggested as a cue to instruct cell polarity orientation, based on overexpression and inhibitor studies in organ cultures [6]. However, there is no PCP phenotype in the cochlea of Wnt7a null mice [6], suggesting that other Wnt proteins function redundantly with Wnt7a. In *Xenopus* and *zebrafish*, Wnt11 and Wnt5 are required for convergent extension movements during gastrulation, which is also regulated by the PCP pathway [7], [8]. However, these Wnts are thought to function permissively in cell polarization, rather than providing a directional cue. The presence of global extrinsic cues that orchestrate polarity orientations has not been shown in any organism.

In *C. elegans*, the Wnt/ß-catenin asymmetry pathway controls asymmetry in most somatic cell divisions occurring along the anterior-posterior axis [9]. In this regulation, Wnt pathway components localize asymmetrically. For example, after asymmetric divisions, the ß-catenin homologs WRM-1 and SYS-1 accumulate in the posterior daughter nuclei, while POP-1/TCF localizes more to the anterior than posterior nuclei [10]. Such localization has been observed in most cell divisions, during which cells are accordingly polarized in the anterior-posterior orientation. But how the polarity orientation is determined is not known, except in a few cases. We have shown that Wnts instructively orient the polarity of the EMS blastomere in embryos and of the T cell in larvae [11]. It has also been suggested that MOM-2/Wnt and LIN-44/Wnt expressed in the anchor cell orient the polarity of the P7.p cell, while EGL-20/Wnt expressed near the anus antagonizes these Wnts to orient the P7.p polarity in the opposite orientation [12]. However, it is not known whether or how Wnts globally regulate the polarity of many other cells.

**Figure 1:**
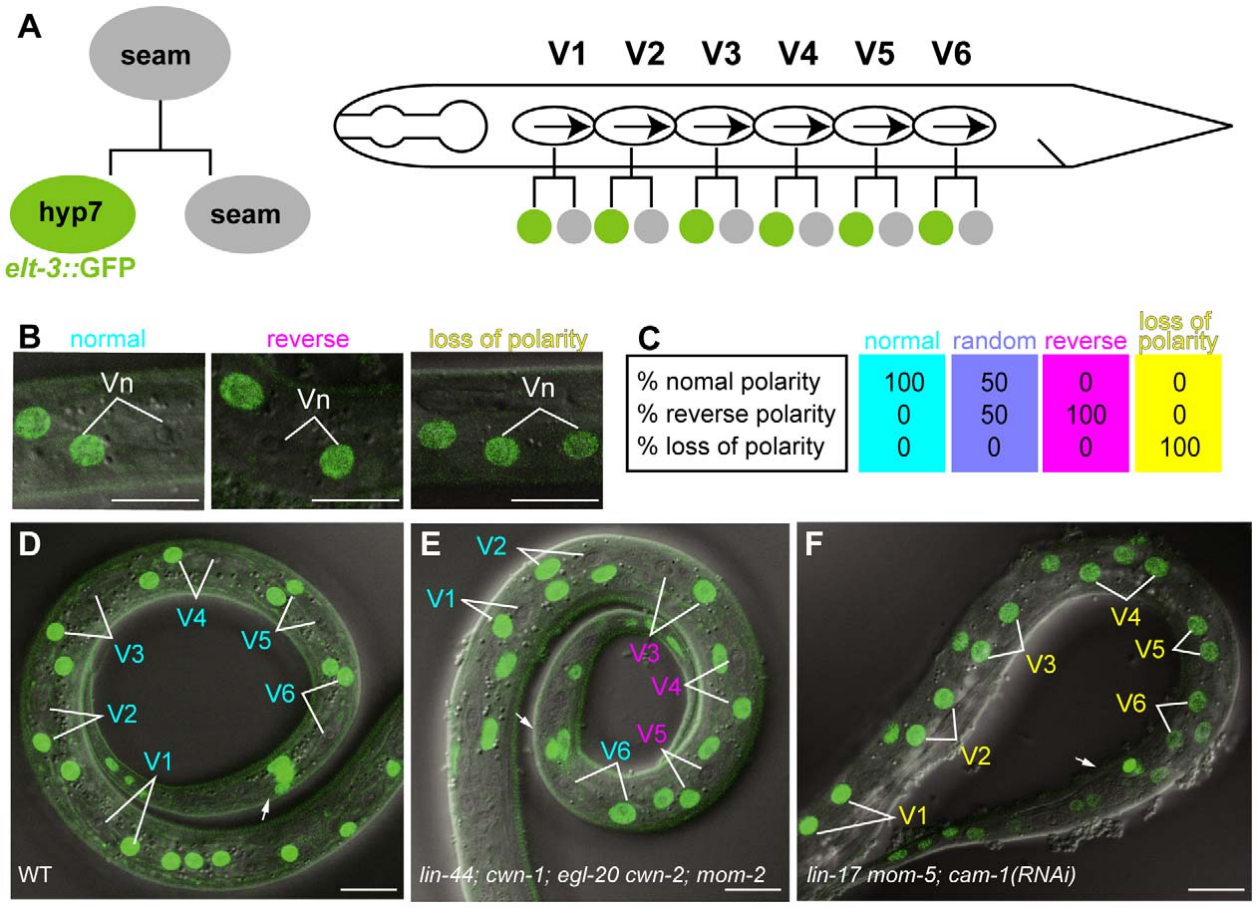
Analyses of seam cell polarity. (A) Schematic drawing of seam cell divisions. Left: Anterior daughter of a seam cell fuses with hyp7 and becomes *elt-3::*GFP-positive. Right: seam cells (V1–V6) on the lateral sides of animals are polarized in the same orientation (represented by arrows) and divide asymmetrically. (B) Examples of the polarity of seam cell (Vn) divisions (normal, reverse, or loss of polarity) as judged by the *elt-3*::GFP expression in the daughter cells. Merged differential interference contrast (DIC) and fluorescent images are shown. (C) An illustration of sample data for the polarity of seam cell divisions for various genotypes. Each colored box represents the polarity of an individual seam cell. Top, middle, and bottom numbers in the boxes are the percentages of individual seam cells with normal, reverse, and loss of polarity, respectively. An RGB color component was assigned for each polarity phenotype (normal = red; reverse = green; loss of polarity = blue), and the color of each box represents the combination of the calculated intensity of each RGB component per seam cell. Intensity was calculated as follows: 255−(% observed phenotype×2.55), where 255 is the maximum intensity of each RGB component, and 2.55 is a factor for standardizing the phenotype percentage to the RGB scale. The colored boxes shown here represent the resulting four standard colors. Cells with 100% normal division-polarity are cyan (red 0, green 255, blue 255); cells with random division-polarity are lavender (red 128, green 128, blue 255); cells with 100% reverse division-polarity are magenta (red 255, green 0, blue 255); and cells with 100% loss of division-polarity are yellow (red 255, green 255, blue 0). In the similar illustrations in the following Figures, intermediate color compositions indicate relative tendencies towards a certain phenotype in relation to these four standards. (D–F) The expression of *elt-3::*GFP at the late L1 stage in wild-type *C. elegans* (D); *lin-44(n1792)*; *cwn-1(ok546)*; *egl-20(n585) cwn-2(ok895)*; *mom-2(ne874ts)*; *vpIs1* (E); and *lin-17(n3091) mom-5(ne12)*; *cam-1(RNAi)*; *vpIs1* (F). The colors of the cell names indicate the polarity of their divisions (normal in cyan, reverse in magenta, and loss of polarity in yellow), as determined by the *elt-3::*GFP expression in their daughter cells. Arrows indicate the anus, which is on the ventral side. Scale bars: 10 µm.

To elucidate the mechanisms of polarity coordination, we focused on a population of epithelial stem cells called seam cells (V cells). At the L1 stage, the six seam cells V1–V6 are positioned on each lateral side of the animals, and repeatedly undergo self-renewing asymmetric cell divisions in each larval stage to produce anterior daughters that fuse with the hypodermal syncytium (hyp7) and posterior daughters that remain as seam cells (Figure 1A) [13]. As with many other cells, the polarity of seam cells is controlled by the Wnt/ß-catenin asymmetry pathway [14], [15], which determines the polarized localization of WRM-1/ß-catenin to the posterior daughter nuclei. Among seam cells, the polarity of the V5 cell reverses fairly frequently in *egl-20*/Wnt mutants [16]. However, Wnt gene regulation of the polarity of other seam cells has not been reported.

By analyzing various compound Wnt mutants, including quintuple Wnt mutants, we found that the Wnt genes *lin-44*, *cwn-1*, *egl-20*, and *cwn-2* are redundantly required to coordinate the orientation of seam cell polarity at the L1 stage, but three of their receptors are essential for generating the cells' polarity in the first place. The Wnt genes are expressed either anterior or posterior to the seam cells, and each one alone can determine the polarity orientation. Our results provide an important basis for elucidating undiscovered mechanisms in the coordination of cell polarity by Wnt genes.

## Results

### Multiple Wnts control seam cell polarity

**Figure 2:**
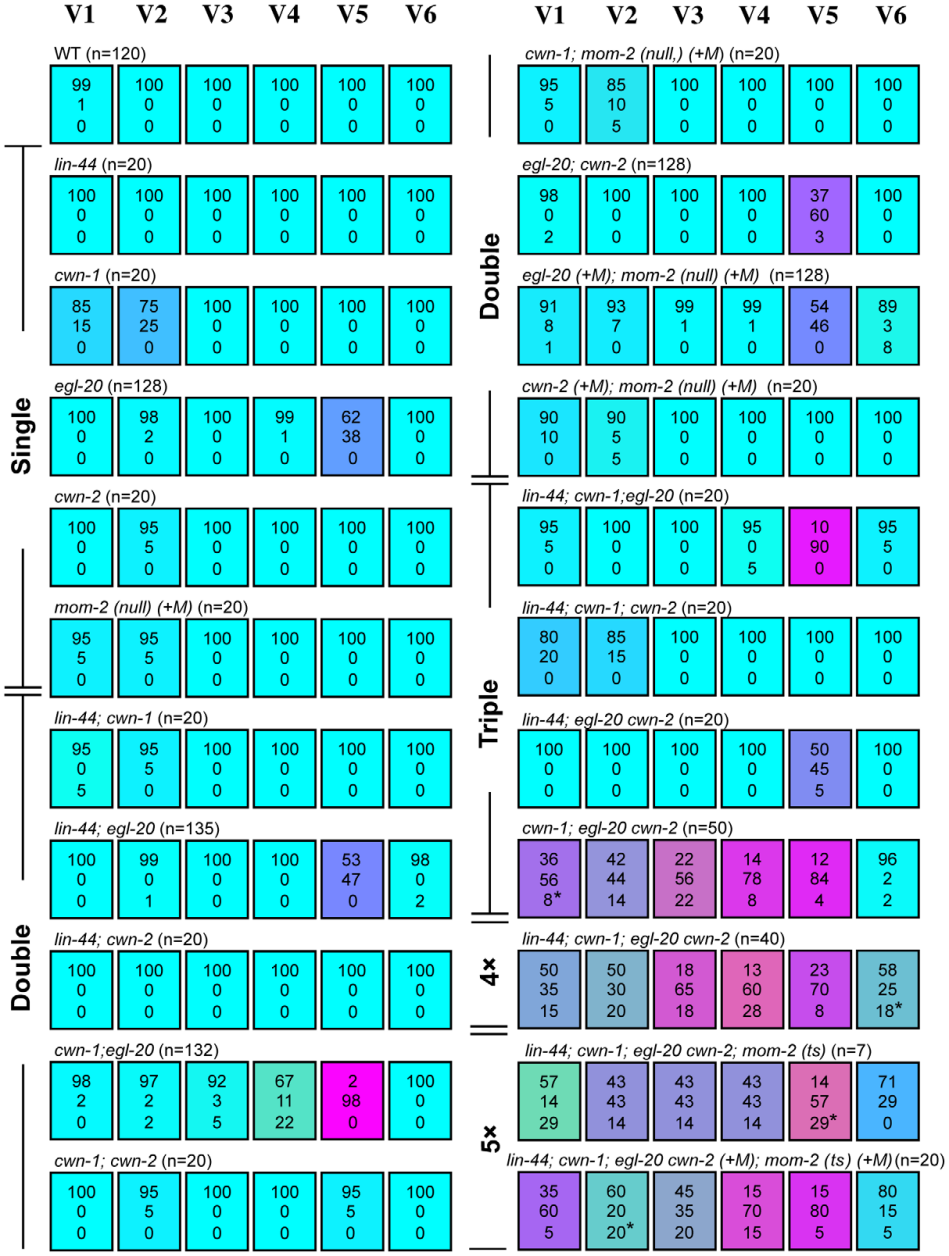
Seam cell polarity orientation is redundantly regulated by multiple Wnts. Each colored box represents the polarity of individual seam cell divisions as in Figure 1C. The symbol (+M) indicates maternal contributions. In most cases, “loss of polarity” indicates divisions in which both daughter cells adopted the hyp7 fate, except for some divisions (indicated by asterisks in this and the following Figures; in all cases, one sample per cell) in which both daughters adopted the seam cell fate. Random asymmetry of the V1–V5 divisions in the *cwn-1*; *egl-20 cwn-2* mutants was also observed using *scm*::GFP (Figure S1).

To analyze the polarity of the seam cell divisions, we used *elt-3*::GFP, which is expressed in hyp7 but not in seam cells [17], [18] (Figure 1A, 1D). About 1 hour after the division of seam cells (V1–V6) at the L1 stage in wild-type animals, the anterior daughters fuse with hyp7; their nuclei immediately begin fluorescing like those of hyp7 cells, because they incorporate GFP from the hyp7 cell. Therefore, we can unambiguously determine the daughter cell fates, from which we can deduce the division polarity type (normal, reverse, or loss of polarity) (Figure 1B). (In Figure 1C and the figures presented below, the proportions of the polarity types of individual seam cells were mathematically converted to RGB colors as described in the figure legend.) The *C. elegans* genome contains five Wnt genes, *lin-44*, *cwn-1*, *cwn-2*, *egl-20*, and *mom-2*. To understand how seam cell polarity is regulated, we first analyzed the phenotypes of animals with mutations in one of the five Wnt genes. Except for *egl-20*, in which the V5 polarity was reversed [16], the Wnt mutants showed weak phenotypes, if any (Figure 2), raising the possibility that multiple Wnt genes redundantly regulate seam cell polarity.

To test this hypothesis, we constructed a strain with mutations in all five Wnt genes (quintuple Wnt mutants). Because a combination of three Wnt null mutations (*cwn-1*, *cwn-2* and *mom-2*) causes complete embryonic lethality [19], we used the temperature-sensitive (ts) mutation *mom-2(ne874)*, in which endoderm production is strongly affected during embryogenesis at restrictive temperatures [20]. Because quintuple Wnt mutants only occasionally reproduce, even at permissive temperatures, we could analyze only 7 animals born from homozygous quintuple Wnt mutants. In addition, we analyzed quintuple mutants from mothers heterozygous for *cwn-2*, *egl-20* and *mom-2*, as shown in Figure 2: *lin-44*; *cwn-1*; *egl-20 cwn-2(+M)*; *mom-2(ts) (+M)*.

We found that the polarity of all the seam cell divisions was abnormal in the quintuple Wnt mutants (Figure 1E and Figure 2, p<0.01 in V1–V6 by Fisher's exact test), indicating that multiple Wnts are redundantly required for appropriately oriented seam cell polarity. Although the phenotypes varied among the cells, the polarity tended to be either normal or reversed, and symmetric division was less frequent (represented by the absence of yellowish colors in Figure 2). Although we cannot exclude residual *mom-2* activity in quintuple mutants with the *mom-2(ts)* allele, the results suggest that seam cells are mostly polarized even in the absence of Wnt functions.

### Most seam cells can be properly polarized by a single Wnt gene

To determine which combinations of Wnt genes are required for the properly oriented polarity of individual seam cells, we analyzed them in double, triple, or quadruple Wnt mutants. The phenotype of quadruple Wnt mutants (*lin-44*; *cwn-1*; *egl-20 cwn-2*) was quite similar to that of quintuple mutants (Figure 2; p>0.1 in V1–V6 for the abnormalities), suggesting that *mom-2* has only minor functions, if any, in seam cell polarity. Next, we constructed triple Wnt mutants from these four Wnt mutations. Through these analyses, we found three distinct regulations that depended on cell type, grouped into V1–V4, V5, and V6.

#### V1–V4

The phenotypes of V1–V4 Wnt triple mutants (*cwn-1*; *egl-20 cwn-2*) were similar to those of Wnt quadruple (p>0.1 in V1–V4 for the abnormalities) and quintuple mutants (p>0.1 in V1, V2 and V4; p>0.05 in V3) (Figure 2), suggesting that the polarity in these cells are regulated primarily by these three Wnt genes. In any double combination of these three Wnt mutations, the polarity of the divisions was almost normal, although V4 was weakly affected by *cwn-1*; *egl-20* (Figure 2) (p<0.01). The results indicate that functions of these three Wnts are redundant in all four of these cells.

#### V6

The most posterior seam cell, V6, was affected in quadruple Wnt mutants (p<0.01), but not in any triple or double combination analyzed (Figure 2). Therefore, the V6 cell polarity is redundantly regulated by the four Wnts.

In summary, V1–V4 and V6 cells are properly polarized by the presence of just one Wnt from among the three Wnts *cwn-1*, *cwn-2* and *egl-20* for V1–V4, or among the four Wnts *lin-44*, *cwn-1*, *cwn-2* and *egl-20* for V6.

#### V5

In contrast to V1–V4 and V6, one Wnt, *egl-20*, is essential for V5 polarity, as reported previously [16]. In *egl-20* mutants, the polarity of the division was reversed in 38% of the V5 cells (Figure 2). This phenotype was strongly enhanced to nearly complete reversal (98%) in *cwn-1*; *egl-20*, suggesting that functions of *cwn-1* and *egl-20* are partially redundant. Although the *cwn-2* mutation slightly enhanced polarity reversal in the *egl-20* background (p<0.01), it instead suppressed the phenotype in the *cwn-1 egl-20* background (p<0.01) (Figure 2), suggesting that *cwn-2*'s functions in the V5 cell polarity are complex. One possibility for the unique regulation of V5 might be its distinct cell lineage compared to the other seam cells. Only the V5 cell produces neurons at the L2 stage. To test this possibility, we analyzed *lin-22* mutants, in which not only V5, but also the V1–V4 cells produce neurons [21]. However, even in *lin-22 egl-20* double mutants, polarity reversal was observed mostly in the V5 cell (data not shown). Therefore, V5's neuron production is unlikely to be the reason for its unique regulation.

### Wnt genes control seam cell polarity through the Wnt/ß-catenin asymmetry pathway

**Figure 3:**
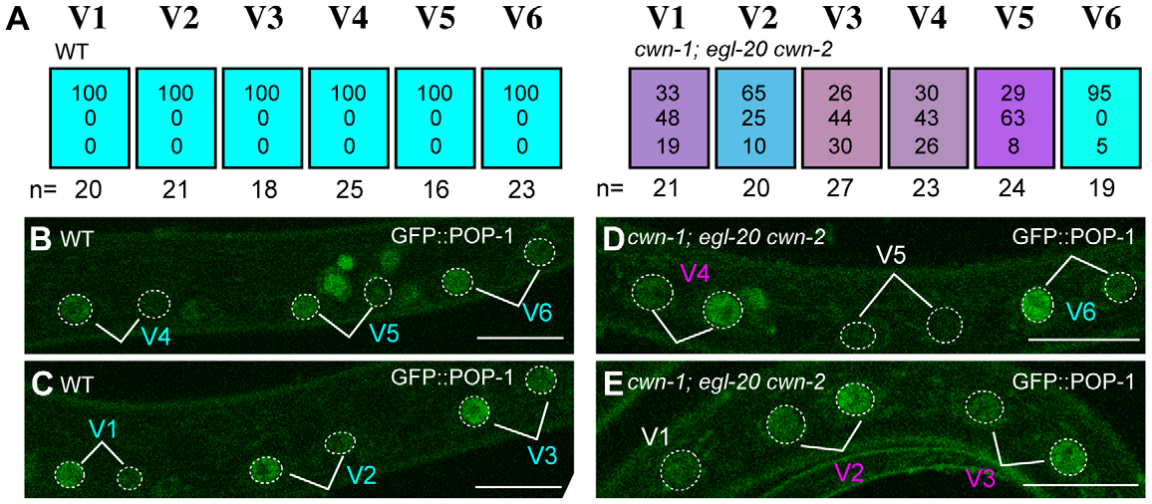
Wnts regulate the Wnt/ß-catenin asymmetry pathway in seam cells. (A) Each colored box represents the polarity of individual seam cell divisions as in Figure 1C, except that polarity was judged by the localization of GFP::POP-1 at the mid-L1 stage. (B–E) Examples of GFP::POP-1 localization in wild-type animals with *qIs74* (GFP::POP-1) (B, C) and in *cwn-1(ok546)*; *egl-20(n585) cwn-2(ok895)*; *qIs74* (D, E). Cell name colors indicate their polarity, as in Figure 1D-1F. GFP::POP-1 localization can be detected for about 1 hour after each seam cell division. Therefore, we can observe polarity of some but not all seam cells in individual animals. For example the V5 cell polarity in (D) could not be judged due to loss of the expression. A V1 cell is shown in (E) before its division. Scale bars: 10 µm.

To confirm that Wnt genes regulate the Wnt/ß-catenin asymmetry pathway, we analyzed POP-1/TCF localization in triple Wnt mutants (*cwn-1*; *egl-20 cwn-2*), in which the polarity of V1–V5 is disrupted. We found that POP-1 asymmetry was abnormal in V1–V5 cells in the triple Wnt mutants (Figure 3A, 3D, 3E; p<0.01 in V1–V5). As judged by *elt-3::*GFP expression (Figure 2), polarity reversal is more frequent than loss of polarity (represented by purplish colors in Figure 3A). Therefore, these Wnt genes control seam cell polarity via the Wnt/ß-catenin asymmetry pathway.

Since seam cells are polarized in a planar (anterior-posterior) orientation in contact with each other before division, interactions between neighboring cells might coordinate their polarity, as with PCP regulation in the *Drosophila* wing. However, in triple Wnt mutants (*cwn-1*; *egl-20 cwn-2*), we did not observe any significant correlation of polarity reversal between neighboring seam cell pairs (data not shown). In addition, the polarity of the V5 cell division is not affected by laser ablation of the V6 cell [16]. Furthermore, we found that the polarity of the seam cell divisions was normal in mutants of the putative PCP components *vang-1*/Van Gogh*(tm1422)* (n = 20) and *prkl-1/*Prickle*(ok3182)* (n = 20) (the phenotype of *vang-1* was analyzed using *scm::GFP*, as described in Materials and Methods). Therefore, it is likely that the polarity of each seam cell is independently controlled by Wnt genes.

### Three Wnt receptors are required for seam cell polarization

To understand how Wnts control polarity, it is important to identify their receptors. The *C. elegans* genome contains six Wnt receptors, four Frizzled (MIG-1, LIN-17, CFZ-2, and MOM-5), one Ror (CAM-1) [22], and one Derailed (LIN-18) family members. Among these, it has been reported that *cam-1*/Ror mutations reverse the polarity of the V1 and V2 cell divisions at a low frequency [23] and that *lin-17*/Frizzled mutants cause mostly symmetric divisions of a tail seam cell called a T cell [24].

**Figure 4:**
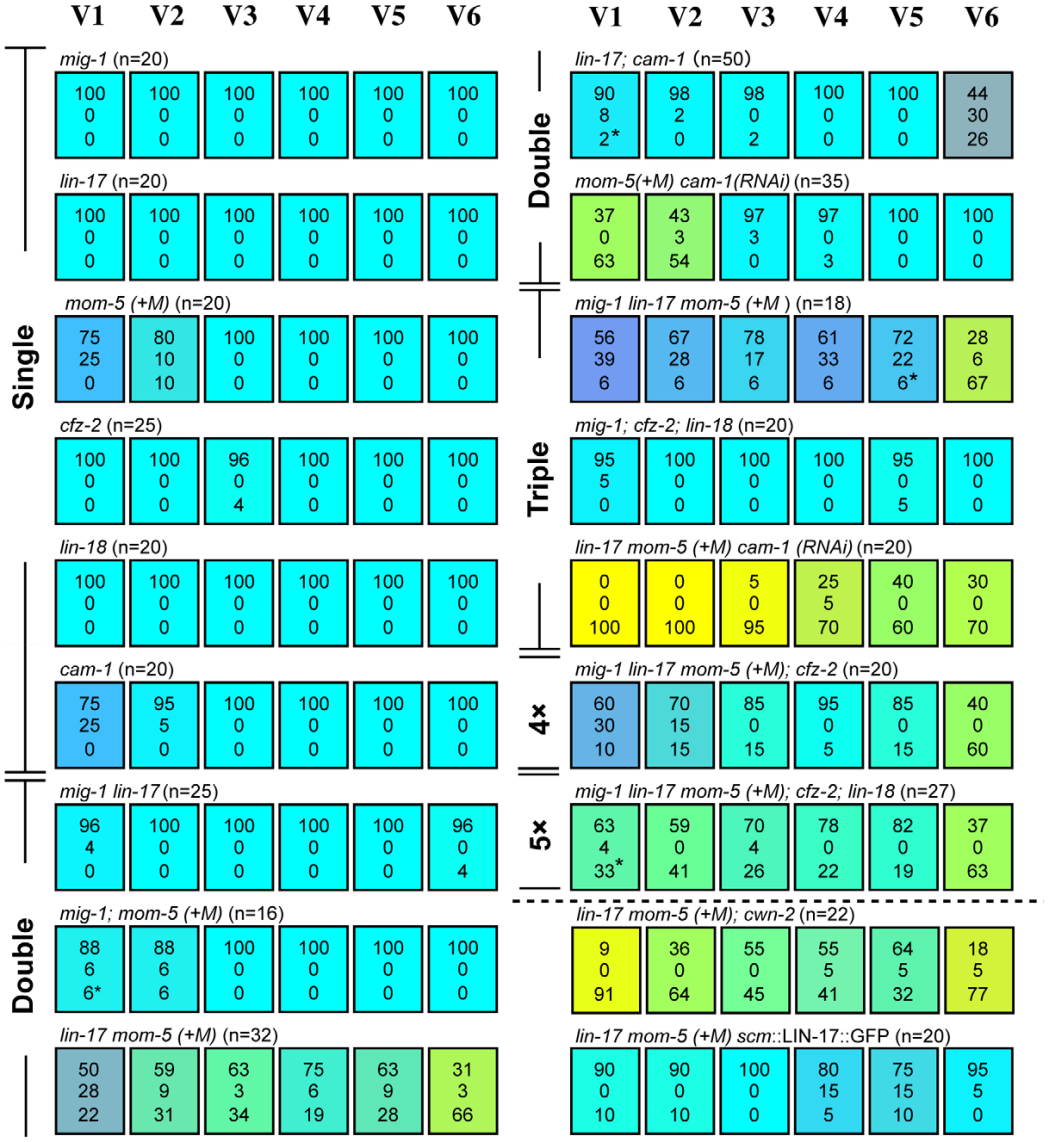
Multiple Wnt receptors redundantly control seam cell polarization. Each colored box represents the polarity of individual seam cell divisions as in Figure 1C; (+M) indicates maternal contributions. Asterisks are as described in Figure 2, legend. Symmetric divisions of the seam cells in *lin-17 mom-5*; *cam-1(RNAi)* mutants were also observed using *scm*::GFP (Figure S1).

First, we analyzed single mutants of each receptor gene. Similar to *cam-1*, the *mom-5* mutation weakly affected the polarity of the V1 and V2 divisions (p<0.01 in V1 and V2). V1–V2 defects were enhanced in *mom-5 cam-1 (RNAi)* animals (p<0.01 in V1 and V2 by the comparison with *mom-5* mutants or *cam-1(RNAi)* animals), indicating that MOM-5 and CAM-1 redundantly control V1–V2 polarity (Figure 4). Single mutants for the other receptors showed only minor defects, if any, in the polarity of seam cell divisions, suggesting that their functions are redundant for V3–V6. Since *lin-17* and *mom-5* show a strong genetic interaction in gonad development [19], we next analyzed *lin-17 mom-5* double Frizzled mutants and found that the polarity of all the seam cell divisions was abnormal (p<0.01 in V1–V6) (Figure 4). The *mig-1*, *cfz-2*, or *lin-18*/Derailed mutations slightly modified the phenotype of the *lin-17 mom-5* mutants. However, since the *mig-1*; *cfz-2*; *lin-18* triple mutants showed nearly normal polarity (Figure 4), these receptors are not essential and are likely to function redundantly with other receptors.

Next, we constructed *lin-17 mom-5*; *cam-1* triple mutants, and found that this combination was embryonically lethal. Therefore, we inhibited *cam-1* by RNAi in *lin-17 mom-5*, and found that all seam cell divisions were symmetric at high penetrance (Figure 1F) (p<0.01 in V1–V6 and p<0.01 in V1–V4, p<0.05 in V5, p>0.1 in V6 for symmetric division by the comparison with wild type and *lin-17 mom-5*, respectively; represented by yellowish colors in Figure 4). These results indicate that LIN-17, MOM-5, and CAM-1 are the main receptors that redundantly regulate seam cell polarity, whereas the receptors MIG-1, CFZ-2, and LIN-18 weakly affect polarity in the absence of the main receptors. Most importantly, the phenotype of *lin-17 mom-5*; *cam-1* is clearly distinct from that of quintuple Wnt mutants in which polarity orientation is randomized (p<0.01 in V1–V6 for symmetric division). These results suggest that Wnt receptors can function even in the absence of Wnts to generate polarity, while Wnts are required to orient polarity.

It was previously suggested that CAM-1 functions as a receptor for CWN-2 [25], [26]. If this is the case for seam cell polarity, the *cwn-2* mutation should have the same or stronger effects than the *cam-1* mutation. However, as described above, the *cwn-2* mutation alone did not affect the V1 cell, which was affected in *cam-1* mutants. Furthermore, the *lin-17 mom-5*; *cwn-2* mutants had a weaker phenotype than *lin-17 mom-5 cam-1(RNAi)* (p<0.05 in V2 and V3, p = 0.066 in V4) (Figure 4). Therefore, it is unlikely that CAM-1 is a specific receptor for CWN-2 for seam cell polarity.

It was reported that *cam-1p*::GFP is expressed in V cells [23]. We found that *lin-17p*::LIN-17::GFP is also expressed in all V cells (Figure S2). To determine whether the receptors functions in seam cells, we expressed LIN-17 specifically in seam cells using the *scm* promoter (*scm*::LIN-17::GFP) [11] and found that the polarity defects in the *lin-17 mom-5* animals were significantly rescued in V1–V3 and V6 (p<0.01 in V1, V3, and V6, p<0.05 in V2, p>0.1 in V4 and V5) (Figure 4), suggesting that at least LIN-17 among the Wnt receptors functions in seam cells.

### Wnts are expressed either anterior or posterior to the V cells

**Figure 5:**
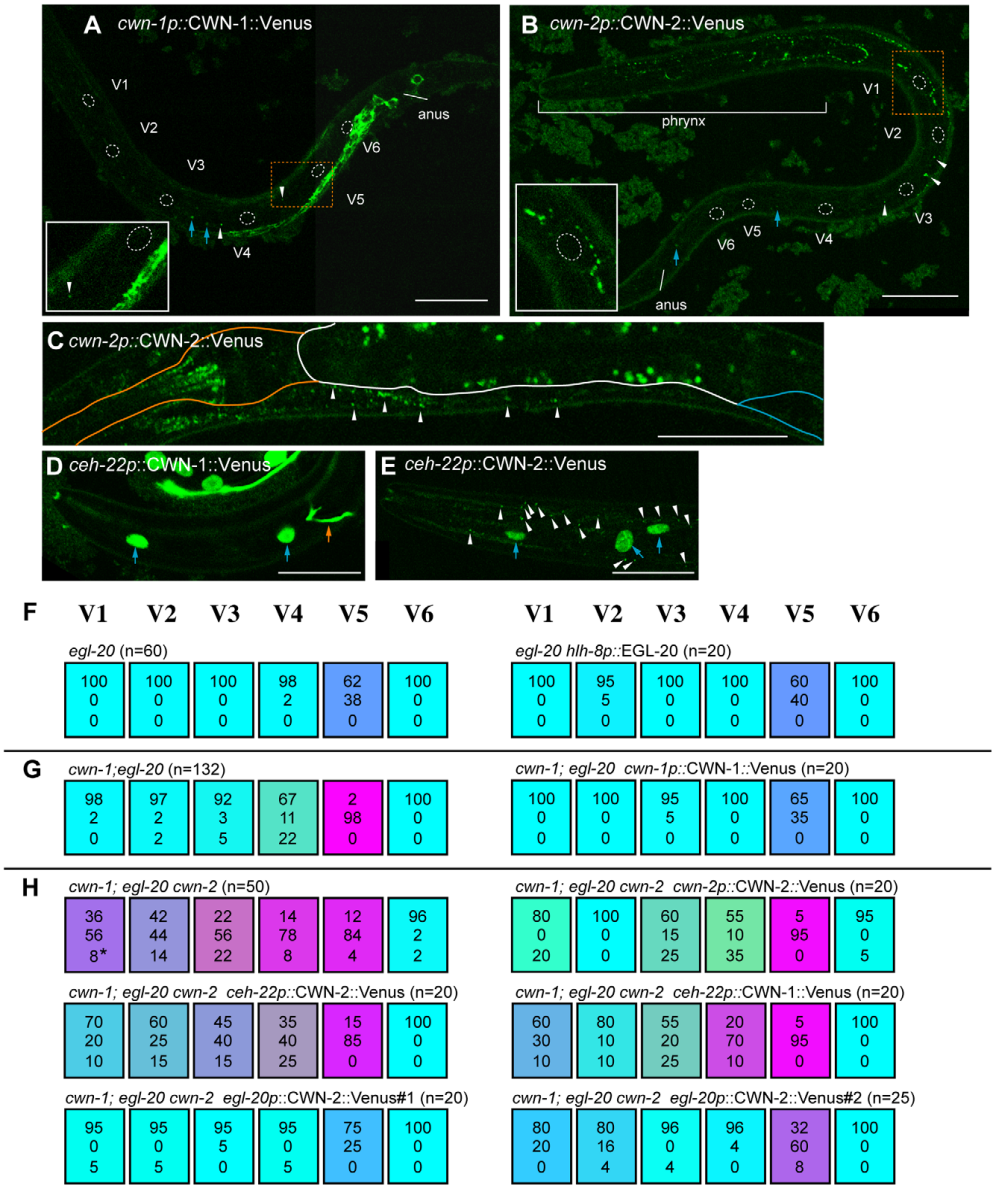
Ectopic Wnt expression rescued compound Wnt mutants. (A–F) The expression of *cwn-1p::*CWN-1::Venus (A), *cwn-2p::*CWN-2::Venus (B, C), *ceh-22p::*CWN-1::Venus (D), and *ceh-22p::*CWN-2::Venus (E). Anterior is to the left and ventral to the bottom, except for (B), in which anterior is to the upper-left and ventral toward the right. Scale bars: 20 µm. Confocal images in (A, B) and (D, E), were focused on hypodermal cells. The focus is on the pharynx/intestine in (C). (A, B) Dashed lines outline seam cell nuclei. White arrowheads and blue arrows indicate some of the GFP puncta and granule autofluorescence, respectively, which were observed even in the corresponding DIC images. Insets show the area indicated by orange dashed boxes, magnified 2-fold; they illustrate the efficient diffusion of *cwn-2p::*CWN-2::Venus but not of *cwn-1p::*CWN-1::Venus. (C) Expression of *cwn-2p::*CWN-2::Venus in the pharynx and near the intestine. Orange, white, and blue lines outline the pharynx, intestine, and gonad, respectively. Puncta of *cwn-2p::*CWN-2::Venus near the intestine (white arrowheads) were observed only in the anterior region. Puncta in the intestine are gut granule autofluorescence. (D, E) Diffusion of *ceh-22p::*CWN-2::Venus (E) but not *ceh-22p::*CWN-1::Venus (D) was observed in the anterior hypodermis near the pharynx. White arrow heads, blue arrows and the orange arrow indicate GFP puncta, hypodermal cells expressing the *elt-3::*GFP marker, and neuronal processes expressing the *mec-4::*GFP marker, respectively. (F–H) Each colored box represents the polarity of individual seam cell divisions, as in Figure 1C. (F) The *egl-20* phenotype was not affected by EGL-20 expression in the M cell (*hlh-8p::*EGL-20). Since the M cell is on the right side of the animals, we scored seam cells only on the right side. (G) *cwn-1p::*CWN-1::Venus rescued the defect of the *cwn-1* mutation in *cwn-1*; *egl-20*. (H) The phenotype of *cwn-1*; *egl-20 cwn-2* was rescued by *cwn-2p::*CWN-2::Venus, by CWN-2 expressed in the pharynx (*ceh-22p::*CWN-2::Venus) or in the cells near the anus (*egl-20p*::CWN-2::Venus#1 and #2 with *mec-4*::GFP and *egl-5*::GFP as coinjection markers, respectively), or by CWN-1 expressed in the pharynx (*ceh-22p::*CWN-1::Venus).

Wnt genes are expressed in specific regions of the animal, either in the anterior (CWN-2) [25], [26] or posterior (LIN-44, EGL-20, and CWN-1) [16], [27]–[29]. In addition, EGL-20 forms a posterior–to-anterior gradient [30]. We examined CWN-1 and CWN-2 protein localization by full-length translational fusion constructs (*cwn-1p::*CWN-1::Venus or *cwn-2p::*CWN-2::Venus). V4 and V5 polarity defects in *cwn-1*; *egl-20* mutants were rescued by *cwn-1p::*CWN-1::Venus (Figure 5G), and V1–V4 defects in *cwn-1*; *egl-20 cwn-2* mutants were partly rescued by *cwn-2p::*CWN-2::Venus (Figure 5H), indicating that these fusion proteins are functional. As reported previously for CWN-1 promoter activity [19], *cwn-1p*::CWN-1::Venus was localized to the cytoplasm and around the cell membrane in posterior muscle cells, both dorsal and ventral (Figure 5A). Although there were variations between animals, the signals clearly tended to be stronger in posterior cells than in the middle of the animal, suggesting that CWN-1 expression may form a gradient.

Consistent with the previous observation that the *cwn-2* promoter is strongly active in the pharynx [25], [26], we detected puncta of *cwn-2p::*CWN-2::Venus mostly around the pharynx. We detected these puncta on the hypodermis, including the seam cells (Figure 5B, white arrowheads), suggesting its diffusion from the pharyngeal region. This is in contrast to *cwn-1p::*CWN-1::Venus, whose diffusion away from its expressing cells was only occasionally observed (white arrowheads in Figure 5A). Although it was also reported that the *cwn-2* promoter is active in the intestine, albeit weaker than in the pharynx [25], [26], we detected *cwn-2p::*CWN-2::Venus puncta only in the anterior region, along the boundary between the intestine and muscle or hypodermis (Figure 5C, white arrowheads). These observations indicate that CWN-2 is mostly distributed to the anterior side of the animal. To confirm that *cwn-2* functions in the pharynx, we used a *ceh-22* promoter to express *cwn-2* in the pharynx of Wnt triple mutants (*cwn-1*; *egl-20 cwn-2*), and found that the phenotype was rescued in V1, V3 and V4 cells (p<0.05 in V1, p<0.1 in V3 and V4, p = 0.14 in V2) (Figure 5H). The weak effects of *ceh-22p::*CWN-2::Venus compared to *cwn-2p:* CWN-2::Venus appear to reflect weaker transgene expression. These results suggest that *cwn-2* is expressed and functions in the pharynx.

### Ectopically expressed Wnts rescued Wnt triple mutants

Our results indicate that each seam cell except V5 can be polarized by a single Wnt gene expressed either anterior or posterior to the cells. For example, V1 is properly polarized merely by *cwn-2* expressed nearby and at its anterior, or by *egl-20* expressed posterior to and far from V1. To determine whether the position of Wnt expression is important in regulating polarity, we expressed Wnt genes ectopically. If Wnts function permissively, abnormal polarity in Wnt compound mutants should probably be rescued irrespective of the location of Wnt expression. If the Wnts were instructive, we expected that ectopic Wnt expression opposite to its normal location would enhance polarity reversals.

As reported previously, EGL-20 expressed in the pharynx by the *myo-2* promoter can rescue V5 polarity defects in *egl-20* mutants [16]. However, since the *myo-2* promoter is also weakly active in the posterior region [11], the appropriate interpretation of these results was uncertain. We first used the *hlh-8* promoter to express *egl-20* in the M cell, a mesodermal blast cell positioned between the V4 and V5 cells on the right side, in *egl-20* mutants [31]. We found that this had no significant effect on V5 cell polarity (Figure 5F), suggesting that *egl-20* does not function (i.e., it is not produced, secreted, or modified) in polarization when it is expressed in the M cell.

We then expressed *cwn-1* or *cwn-2* ectopically in the anterior (using the *ceh-22* promoter) [32], [33] posterior (using the *egl-20* promoter) [29] regions in Wnt triple mutants (*cwn-1*; *egl-20 cwn-2*). Surprisingly, the posterior expression of CWN-2, which is normally expressed in the pharynx, efficiently rescued the triple mutant phenotype (Figure 5H, p<0.01 in V1–V5). Similarly, the anterior expression of CWN-1, which is normally expressed in the posterior region, appeared to rescue the polarity defects of the V1–V3 divisions (Figure 5H, V1 p = 0.1076, V2 p<0.01, V3 p<0.05). The effect of *ceh-22p*::CWN-1::Venus was comparable to that of *ceh-22p*::CWN-2::Venus. These results seem to suggest that the position of Wnt expression is not important and that Wnt functions are not instructive, even though Wnts are required for correct polarity orientation. However, the results can also be explained by assuming that functions of Wnts are determined by the cells that express them (see Discussion).

It is noteworthy that, even though *ceh-22p*::CWN-1::Venus and *ceh-22p*::CWN-2::Venus express these Wnts from the same *ceh-22* promoter, we detected puncta of CWN-2::Venus but not CWN-1::Venus outside of the pharynx (white arrowheads in Figure 5D, 5E). Together with the efficient diffusion of *cwn-2p*::CWN-2::Venus but not *cwn-1p*::CWN-1::Venus described above (Figure 5A and 5B), the results suggest that these Wnts have distinct diffusion properties. Because *ceh-22p*::CWN-1::Venus rescued V1–V3 polarity, CWN-1 is likely to be diffused, but at such low levels that it was undetectable. The difference may reflect CWN-1's lower diffusion or weaker ability to form puncta as compared to CWN-2.

### Wnt-independent regulation of somatic gonad precursor polarity

**Figure 6:**
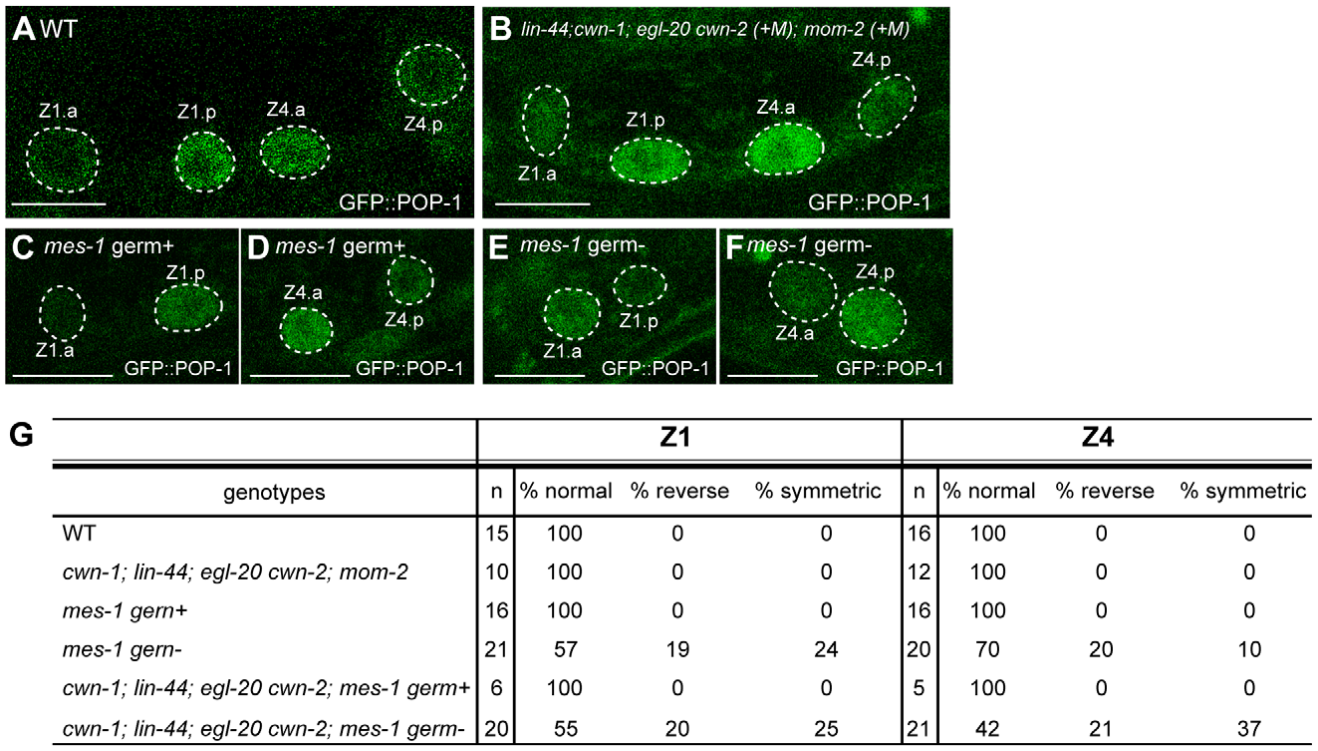
Regulation of SGP polarity. (A–F) GFP::POP-1 localization in SGP daughters in wild-type (A), quintuple Wnt mutants (+M) (B), *mes-1* mutants with germ cells (C, D), and *mes-1* mutants without germ cells (E, F). Anterior is to the left. Scale bars: 10 µm. (G) The table summarizes the GFP::POP-1 expression data.

Similar to seam cells, the Wnts regulating the polarity of Z1 and Z4 cells, which are somatic gonad precursors (SGPs), have not been identified. The SGPs have a mirror-symmetric polarity, which is important for producing the mirror symmetry of the *C. elegans* gonad [34]. POP-1 asymmetry in the Z1 daughters is reversed compared to other cells, including Z4. POP-1 is higher in the posterior and anterior daughters of Z1 and Z4, respectively (Figure 6A, 6G) [35]. SGP polarity is also regulated by the Wnt/ß-catenin asymmetry pathway [35], although the involvement of Wnt genes has not been demonstrated. We found that the SGP polarity was not affected in quintuple Wnt mutants from mothers heterozygous for *cwn-2*, *egl-20* and *mom-2*, as judged by the normal POP-1 localization (Figure 6B, 6G) and the presence of distal tip cells (DTCs; data not shown). Although we could not analyze the POP-1 asymmetry in the quintuple Wnt mutants from homozygous mothers, all such animals we examined (n = 85) had two gonad arms as in wild type, indicating that normal numbers of DTCs were produced from SGPs. These results suggest that the polarity of SGPs is regulated by Wnt-independent mechanisms.

To explore the polarity-regulating mechanisms in SGPs, we used *mes-1* mutants, which frequently lack germ cells [36], to analyze the roles of the germ cells Z2 and Z3, which are positioned between Z1 and Z4. In *mes-1* mutants lacking germ cells, the polarity of both Z1 and Z4 was abnormal, although the defect in Z4 was weaker than that in Z1 (Z1 p<0.01, Z4 p<0.05) (Figure 6E, 6F, 6G). Such defects were not observed in *mes-1* mutants that had germ cells (Figure 6C, 6D, 6G). These results suggest that non-Wnt signals from germ cells control SGP polarity and hence regulate the proper organization of the somatic gonad.

We also examined the possibility that these germ cell signals function redundantly with Wnts. In quadruple Wnt mutants (*lin-44*; *cwn-1*; *egl-20 cwn-2*) lacking germ cells due to the *mes-1* mutation, polarity defects appear to be enhanced in Z4 but not Z1 as compared to *mes-1* mutants, although the difference did not reach significance (Figure 6G) (Z1: *p* = 1.0, Z4: *p* = 0.11), raising the possibility that Z4 polarity is redundantly controlled by Wnts and signals from germ cells. In contrast, the polarity of the Z1 cell appeared not to be affected by the Wnt mutations, and Z1, in wild type, exhibits a reversed orientation compared with Z4 and the seam cells (i.e., POP-1 is higher in the posterior daughter). Z1 may therefore be regulated by signals from germ cells but may be insensitive to Wnt signals.

## Discussion

### Redundant regulation by multiple Wnts

We have shown that seam cell polarity is redundantly regulated by multiple Wnt genes. The V1–V4 and V6 cells are affected only by combinations of three and four Wnt mutations, respectively. Such redundancy has been reported in other organisms [37]. For example, double knockout of Wnt1 and Wnt3a in mice causes much stronger CNS developmental abnormalities than the single knockouts [38]. Because all metazoan species have multiple Wnt genes (e.g., 19 in humans), our results suggest that Wnt genes in any organism may have undiscovered functions that can not be identified by the inhibition of one or a few of them.

### Distinct regulation of polarity orientation and polarity generation

The defects observed in Wnt mutations in any combination were mostly randomized (normal or reverse) polarity, and less frequently, loss of polarity. Similar observations were reported in a mutant lacking *mig-14*/Wntless function, which is required for Wnt secretion [39]. Our observations are consistent with a recent report that seam cell numbers are not significantly altered in *lin-44*; *cwn-1*; *egl-20 cwn-2* animals [15], since the cell numbers were not affected by random orientations of their asymmetry. Even though quintuple Wnt mutants may contain residual *mom-2* activity from the ts allele, our results strongly suggest that functions of at least four Wnts (*lin-44*, *cwn-1*, *cwn-2*, and *egl-20*) determine the polarity orientation of seam cells. In contrast, cell polarization itself appeared to be Wnt-independent, although we cannot eliminate the possibility that cells were not polarized in the complete absence of Wnt functions.

In contrast to the randomized polarity found in compound Wnt mutants, triple receptor mutants (*lin-17 mom-5*; *cam-1*) showed a severe loss of polarity. These three receptors are likely to function in the polarity generation that occurs even in the absence of Wnts. Even though the other three receptors (MIG-1, CFZ-2, and LIN-18) appear to be involved in regulating polarity, based on genetic interactions with *lin-17 mom-5* mutations, their triple mutants showed nearly normal polarity. Therefore, it is likely that LIN-17, MOM-5, and CAM-1 function in the regulation of polarity orientation as Wnt receptors in addition to having a role in the polarity generation that occurs even in the absence of Wnts, although their activities may be modified by the other three receptors (MIG-1, CFZ-2, and LIN-18). Consistent with this interpretation, strains with mutations in these three receptors showed polarity reversal: V1 and V2 in *cam-1* or *mom-5* single mutants, and V6 in *lin-17 cam-1* double mutants. Our results strongly suggest the presence of distinct mechanisms for polarity orientation, which is Wnt-dependent, and polarity generation, which can occur independently of Wnts.

### Do Wnts permissively control polarity orientation?

**Figure 7:**
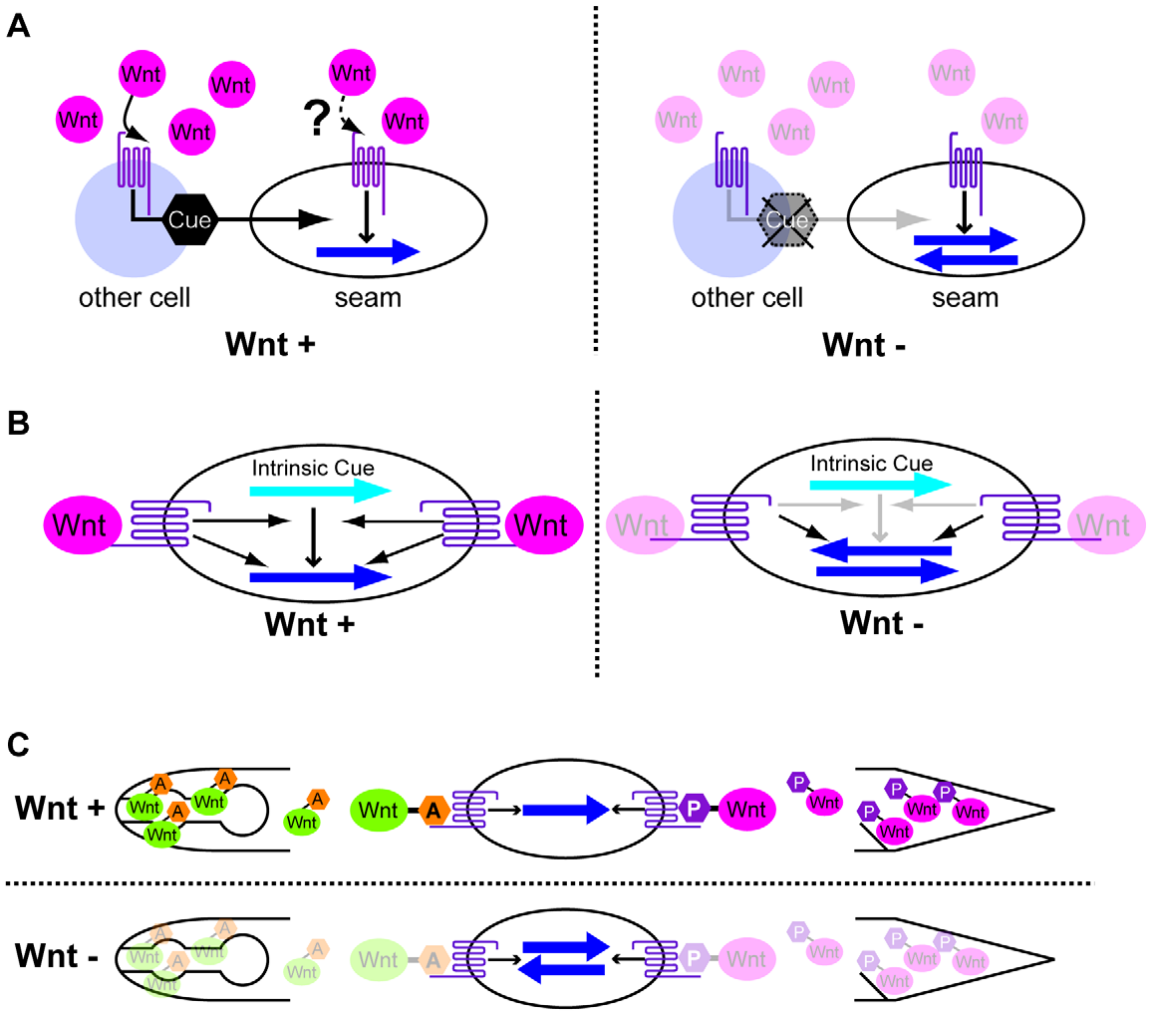
Possible models for polarity orientation by Wnts. (A) Wnts activate the production of polarity cues (black hexagons) through their receptors in cells other than seam cells. The Wnt receptors also function in polarity generation in seam cells using the cues but not Wnts as directional information. (B) Wnts and their receptors function in the interpretation of intrinsic polarity cues (light blue arrows). The receptors also function in polarity generation even in the absence of Wnts. (C) Polarity is instructively oriented by Wnts that are modified by Wnt-expressing cells. That is, anteriorly and posteriorly expressed Wnts receive “anterior modification” (orange hexagons A) and “posterior modification” (magenta hexagons P), respectively. Wnt receptors recognize the modifications to properly orient the polarity. In all the panels, the dark blue arrows show the polarity orientation that is randomized in the absence of Wnts. Anterior is to the left.

Our ectopic expression experiments appear to indicate that although Wnt functions are required to correctly orient polarity, those functions are permissive. Assuming Wnts are permissive, how do they control polarity orientation in seam cells? One model is that Wnts act indirectly through other cells that produce real polarity cues in response to Wnts (Figure 7A). In this case, the same Wnt receptors should function in other cells to produce the cues, and in seam cells to generate polarity. For this model, it is strange that, even though Wnts are apparently present near the seam cells, the Wnt receptor activity to polarize seam cells appears not to be affected by Wnts. Together with our finding that LIN-17 functions in seam cells, this model appears unlikely.

A second model is that Wnt receptors function only in seam cells. They have two distinct functions: one to generate polarity via the Wnt/ß-catenin asymmetry pathway, and the other to interpret intrinsic polarity cues (which might be determined by extrinsic cues) through an unknown pathway (orientation pathway) to generate polarity orientation–but only when they are activated by Wnts (Figure 7B). In the absence of Wnts, the receptors still function to polarize cells, but the intrinsic cues cannot be used, resulting in randomly oriented polarity. Although BAR-1/ß-catenin, which functions downstream of LIN-17 in the migration of the Q neuroblast [40], appears to be a good candidate for mediating the orientation pathway, *bar-1* single mutants have normal seam cell divisions (H.S. unpublished observation). Whatever the mechanism of the orientation pathway is, the key question regarding this model is how Wnts elicit the function of the receptors to activate the orientation pathway without affecting the receptors' function in the Wnt/ß-catenin asymmetry pathway, which generates polarity even in the absence of Wnts.

### Possible Wnt instructive functions in polarity orientation

Because Wnts instruct the polarity of some cells (EMS, T, and P7.p) [11], [12], it is reasonable to imagine that Wnts also instruct seam cells. Assuming that Wnts are instructive, how are the results of ectopic expression explained? One model would be that Wnts' functions depend on the cells that express them. For instance, CWN-2, which is expressed in the pharynx, might receive some specific modification, say, “anterior modification,” whereas CWN-1, which is expressed in the posterior region, might receive a different modification, say, “posterior modification” (Figure 7C). When cells receive CWN-2 with the anterior modification from their anterior side, they recognize the direction of the Wnt source as “anterior” and localize their signaling components accordingly (e.g. POP-1 in the anterior daughter nuclei). When CWN-1 is ectopically expressed in the pharynx, it may receive anterior modification, like CWN-2, and function like CWN-2 to instruct normal seam cell polarity, rather than functioning like CWN-1 with posterior modification.

This model can explain EGL-20's lack of function when expressed in the M cell–assuming that the M cell cannot modify EGL-20. In addition, we have reported that LIN-44 expressed by the *egl-5* promoter (*egl-5*::LIN-44) anterior to a T cell can efficiently reverse T cell polarity in the absence of endogenous LIN-44 expressed at the posterior of the T cell [11]. However, in the presence of endogenous LIN-44 (LIN-44 is expressed in both sides of the T cell), the effect of *egl-5*::LIN-44 is quite weak despite *egl-5*'s promoter activity being stronger, as judged by *egl-5*::GFP, than that of the *lin-44* promoter, as judged by *lin-44*::GFP. This observation is also consistent with the model that Wnt functions depend on the cells that express it. Another possibility for cell-specific Wnt functions is that Wnt-expressing cells or their neighbors express specific cofactors of Wnts that bind tightly to Wnts and determine their functions.

Even though there is no direct evidence for the above models, and other explanations may be possible, our results suggest the presence of novel mechanisms that control the orientation of cell polarity. Such mechanisms, as well as the redundancy of Wnt proteins, may also explain Wnt functions that control cell polarity in other organisms.

## Materials and Methods

### Strains, cloning, culture, and RNAi

N2 Bristol was used as the wild-type strain [41]. The animals were cultured at 22.5°C, except for strains containing *mom-2 (ne874ts)* [20]. The following alleles were used: *lin-44(n1792)* (nonsense) [42]; *cwn-1(ok546)* (deletion) [43]; *cwn-2(ok895)* (deletion) [43]; *egl-20(n585)* (missense, but behaves like null) [44]; *mom-2(or309)* (deletion) [45]; *mom-2(ne874ts)* (missense); *lin-17(n3091)* (nonsense) [24]; *mig-1(e1787)* (nonsense) [28]; *mom-5(ne12)* (nonsense) [46]; *cam-1(gm122)* (nonsense) [23]; *cfz-2(ok1201)* (deletion) [43]; *lin-18(e620)* (nonsense) [47]; *mes-1(bn7)* [48]; *vang-1(tm1422)* (deletion) [49]; *prkl-1(ok3182)* (deletion); and *lin-22(n372)* (missense) [50]. Molecular information of *cwn-1(ok546)*, *cwn-2(ok895)*, *mom-2(or309) cfz-2(ok1201)*, *vang-1(tm1422)* and *prkl-1(ok3182)* is described in http://www.cbs.umn.edu/CGC/index.html.

The genotypes of compound strains were confirmed either by PCR (*cwn-1*, *cwn-2*, *cfz-2*, *vang-1*, and *prkl-1*), sequencing (*lin-44*, *egl-20*, *mom-2(ne874ts)*, *mig-1*, *cam-1*, and *lin-18* ), or by their phenotype (Psa for *lin-44*, maternal effect lethal for *mom-2(or309)* and *mom-5*, bivulva for *lin-17*, and maternal effect sterile for *mes-1*). The strains containing *mom-5(ne12) mom-2(or309)* were maintained as heterozygotes over *hT2[qIs48]* and *nT1[qIs51]*, respectively, which are marked by GFP expression. Non-fluorescent homozygotes were analyzed for their phenotype. The quintuple Wnt mutants were maintained at 15°C as *lin-44*; *cwn-1*; *cwn-2 egl-20/nT1[qIs51]*; *mom-2/nT1*. The phenotype was analyzed in non-fluorescent homozygotes or their progeny, which were shifted to 25°C during late embryogenesis. RNAi for *cam-1* was performed by feeding RNAi (Ahringer Lab RNAi protocol, http://www.gurdon.cam.ac.uk/~ahringerlab/pages/rnai.html) using the RNAi clone I-6L11.

### Analyses of seam cell phenotypes

In most cases, the polarity of seam cell divisions was analyzed using *elt-3::*GFP *(vpIs1)* [18] expressed in hyp7, except for *cfz-2* single mutants, which were analyzed by *scm::GFP (wIs51)* [51]; *lin-18* single mutants, analyzed by *ajm-1::*GFP (*ncIs13*) [52]; *vang-1*, analyzed by *wIs51*; and compound strains with *lin-18* mutations, also analyzed by *wIs51*. GFP markers, including GFP::POP-1 *(qIs74)* [53], *cwn-1p::*CWN-1::Venus, and *cwn-2p::*CWN-2::Venus, were analyzed by confocal microscope (Zeiss LSM510). Statistical analysis was performed with the Fisher exact test.

### Plasmid construction

*cwn-1p*::CWN-1::Venus and *cwn2p*::CWN-2::Venus were constructed from PCR fragments containing their promoter regions (1.8 kb and 6.1 kb, respectively), and the entire coding regions were amplified by PCR from the fosmids WRM0620cE04 and WRM0622bE06, respectively, inserted into a pPD95.75::wVenus derived from pPD95.75 (a gift from A. Fire) and containing the Venus gene optimized for *C. elegans* codon usage in place of the GFP gene. The plasmids *ceh-22p*::CWN-1::Venus and *ceh-22p*::CWN-2::Venus contain a *ceh-22* promoter fragment from pCW2.1 [32] and full-length cDNAs (yk236a10 and yk343h8, respectively) inserted into pPD95.75::wVenus. For *egl-20*p*::*CWN-2::Venus, a 6.8 kb *egl-20* promoter fragment and yk343h8 were inserted into pPD95.75::wVenus. The plasmids were injected as described previously [54], with pBlueScript SK+ DNA and the co-injection markers *unc-76*-rescuing plasmids for *cwn-1p*::CWN-1::Venus and *cwn2p*::CWN-2::Venus injected into *unc-76(e911)* for expression analyses; *ceh-22p*::GFP for the *cwn-1*p::CWN-1::Venus rescue experiment; and *mec-4*::GFP [55] for *ceh-22p*::CWN-2::Venus, *ceh-22p*::CWN-1::Venus, and the *cwn-2p*::CWN-2::Venus rescue experiment. Either *mec-4*::GFP or *egl-5*::GFP [11] was used for *egl-20p*::CWN-2::Venus. The *hlh-8p*::EGL-20 plasmid contains a 1.3 kb PCR fragment just upstream of the start codon of the *hlh-8* gene from a cosmid C02B8 and an *egl-20* cDNA (yk1183a10) subcloned into pPD49.26 (a gift from A. Fire). *lin-17p*::LIN-17::GFP was constructed by inserting a HindIII-KpnI fragment of pSH6 [24] into pPD95.77 (a gift from A. Fire).

## Supporting Information

Figure S1 Seam cell defects in triple Wnts and triple receptor mutants. This phenotype was analyzed using *elt-3*::GFP (left) or *scm*::GFP (right). Each colored box represents the polarity of individual seam cell divisions as in Figure 1C; (+M) indicates maternal contributions. Asterisks are as described in the legend for Figure 2.
(TIF)

Figure S2 The expression of *lin-17p*::LIN-17::GFP in the V cells. Anterior is to the left; ventral is to the bottom. Shapes of the nuclei are indicated by dotted lines. Scale bars: 10 µm.
(TIF)
