# Supplementary figures and images for: Multiple Wnts Redundantly Control Polarity Orientation in Caenorhabditis elegans Epithelial Stem Cells
Source: PLoS Genet. 2011 Oct 13;7(10):e1002308. doi: 10.1371/journal.pgen.1002308 (PMC3192832; doi:10.1371/journal.pgen.1002308)

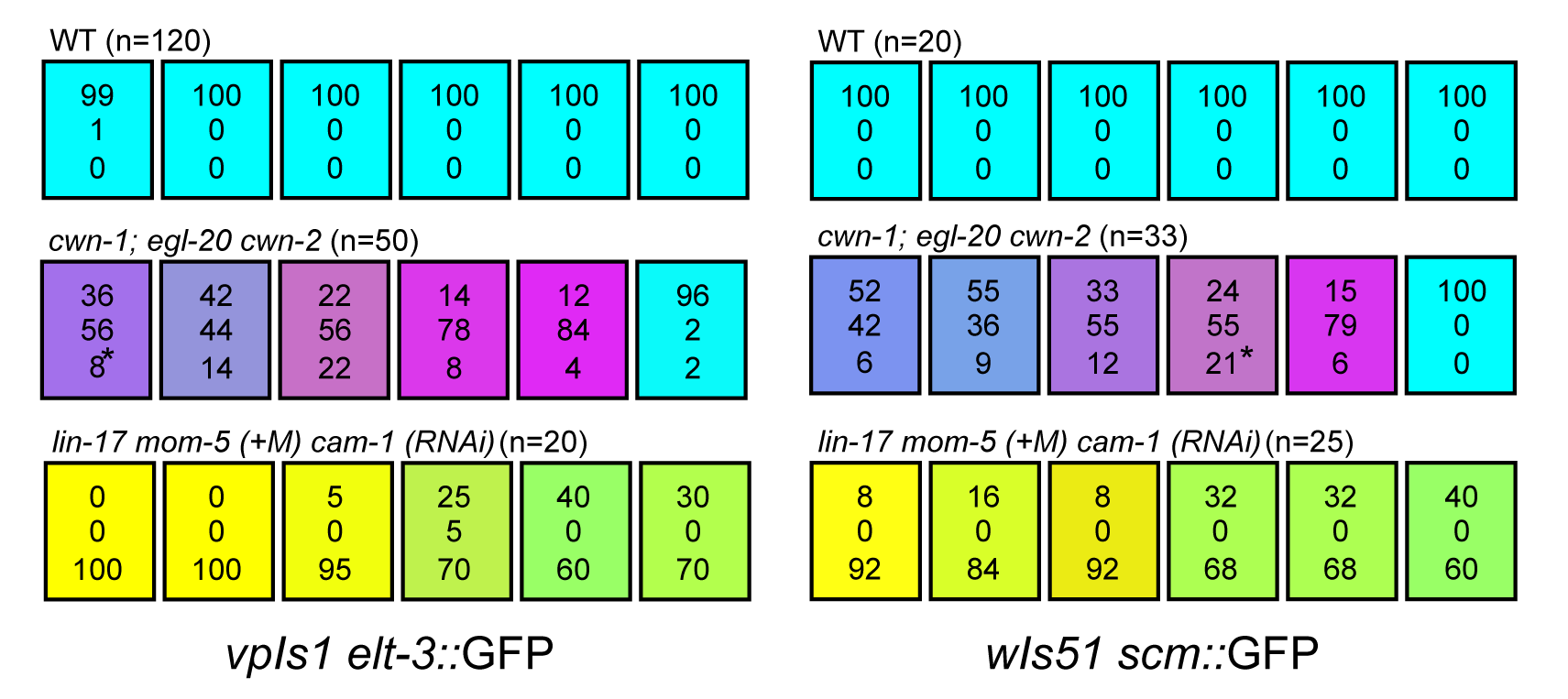

Supplement: Figure S1 — Seam cell defects in triple Wnts and triple receptor mutants. This phenotype was analyzed using elt-3::GFP (left) or scm::GFP (right). Each colored box represents the polarity of individual seam cell divisions as in Figure 1C; (+M) indicates maternal contributions. Asterisks are as described in the legend for Figure 2. (TIF) [file pgen.1002308.s001.tif]

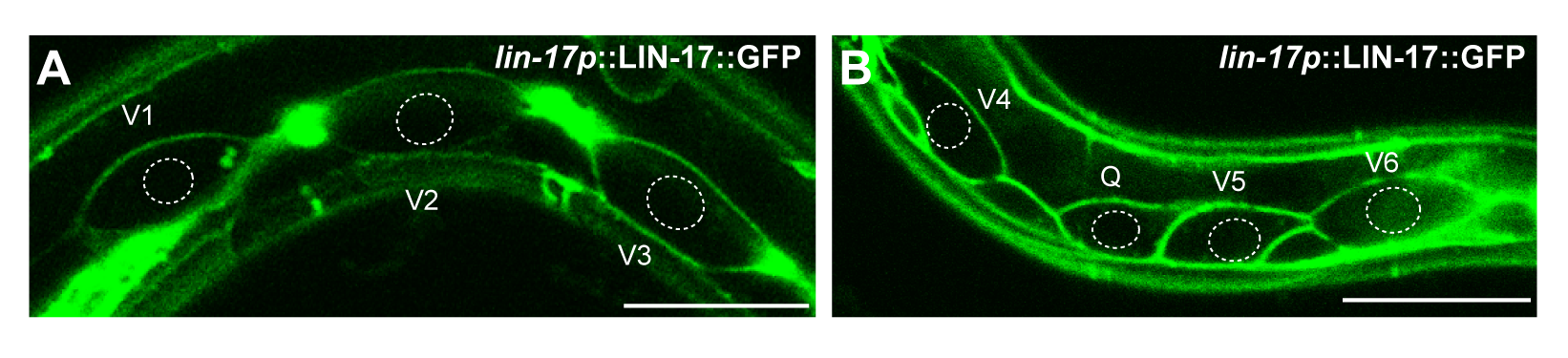

Supplement: Figure S2 — The expression of lin-17p::LIN-17::GFP in the V cells. Anterior is to the left; ventral is to the bottom. Shapes of the nuclei are indicated by dotted lines. Scale bars: 10 µm. (TIF) [file pgen.1002308.s002.tif]
